# Bilateral Empyema in Lemierre’s Syndrome Due to Streptococcus pyogenes: An Atypical Presentation in an Immunocompromised Patient

**DOI:** 10.7759/cureus.118608

**Published:** 2026-10-26

**Authors:** Amy Barden, Archana Balakrishnan, Namuna Sharma

**Affiliations:** 1 Hospital Medicine, Eastbourne District General Hospital, Eastbourne, GBR

**Keywords:** bilateral empyema, immunocompromised, jugular vein thrombophlebitis, lemierre's syndrome (ls), septic embolism, streptococcus pyogenes infection

## Abstract

Lemierre’s syndrome (LS) is a rare yet severe condition that usually develops as a complication of oropharyngeal infection. Infection extends into the parapharyngeal space with subsequent septic thrombophlebitis of the internal jugular vein (IJV), which can result in septicaemia and metastatic lesions. Diagnosed cases of LS have significantly decreased with the introduction of antibiotics; however, several studies report ongoing incidence in recent years. The syndrome usually affects young, healthy individuals, and there is still an association with significant morbidity and mortality. In this report, we discuss an interesting case of a 56-year-old immunocompromised woman who presented with a parapharyngeal abscess and subsequently developed IJV thrombosis and bilateral multiloculated empyema requiring thoracoscopic debridement. The identified causative organism was *Streptococcus pyogenes*. There are few documented cases of LS in immunocompromised individuals or with *Streptococcus* organisms, as identified in this case. This presentation highlights the requirement of prompt imaging, intervention, and escalation of treatment for successful outcomes.

## Introduction

First classified by French physician Andre Lemierre in 1936, Lemierre’s syndrome (LS) is classified by four criteria: (1) acute oropharyngeal infection or abscess, (2) septicaemia, (3) thrombophlebitis of the internal jugular vein (IJV), and (4) secondary metastatic abscesses, most commonly to the lungs and joints [1,2]. The most common causative organism is *Fusobacterium necrophorum* (*F. necrophorum*); however, a recent systematic review found several case reports describing alternative causative agents including *Streptococcus* species and *Staphylococcus aureus* [3,4].

The mainstay of treatment is prompt initiation of antibiotic therapy, tailored to culture results as soon as available. Further surgical management in cases of abscess formation or respiratory distress may be required [4-6]. Current evidence surrounding anticoagulation is controversial, with its use not validated by controlled trials, and the literature suggests antibiotics remain the gold standard of treatment, with individuals recovering well without therapeutic doses of anticoagulation [7,8]. However, in patients with high thrombotic burden, multiorgan disease, or failure to respond to initial therapy, it may be recommended [9]. Herein, we present the case of an immunocompromised individual who developed IJV thrombosis and multiloculated, bilateral empyema following oropharyngeal infection with an unusual causative organism.

## Case presentation

This 56-year-old woman presented to the emergency department with a three-day history of sore throat, fever, and submandibular swelling. Her past medical history included rheumatoid arthritis treated with adalimumab. Computed tomography (CT) imaging revealed an extensive neck abscess extending from the tonsillar regions inferiorly into the superior mediastinum, complicated by thrombosis of the right IJV (Figure 1). Laboratory results displayed leucocytosis (22.8 × 10⁹/L) and raised C-reactive protein (CRP) (565 mg/L); full results are in Table 1. Pus cultures grew *Streptococcus pyogenes* (*S. pyogenes*), and she was commenced on intravenous (IV) ceftriaxone and metronidazole.

**Figure 1:**
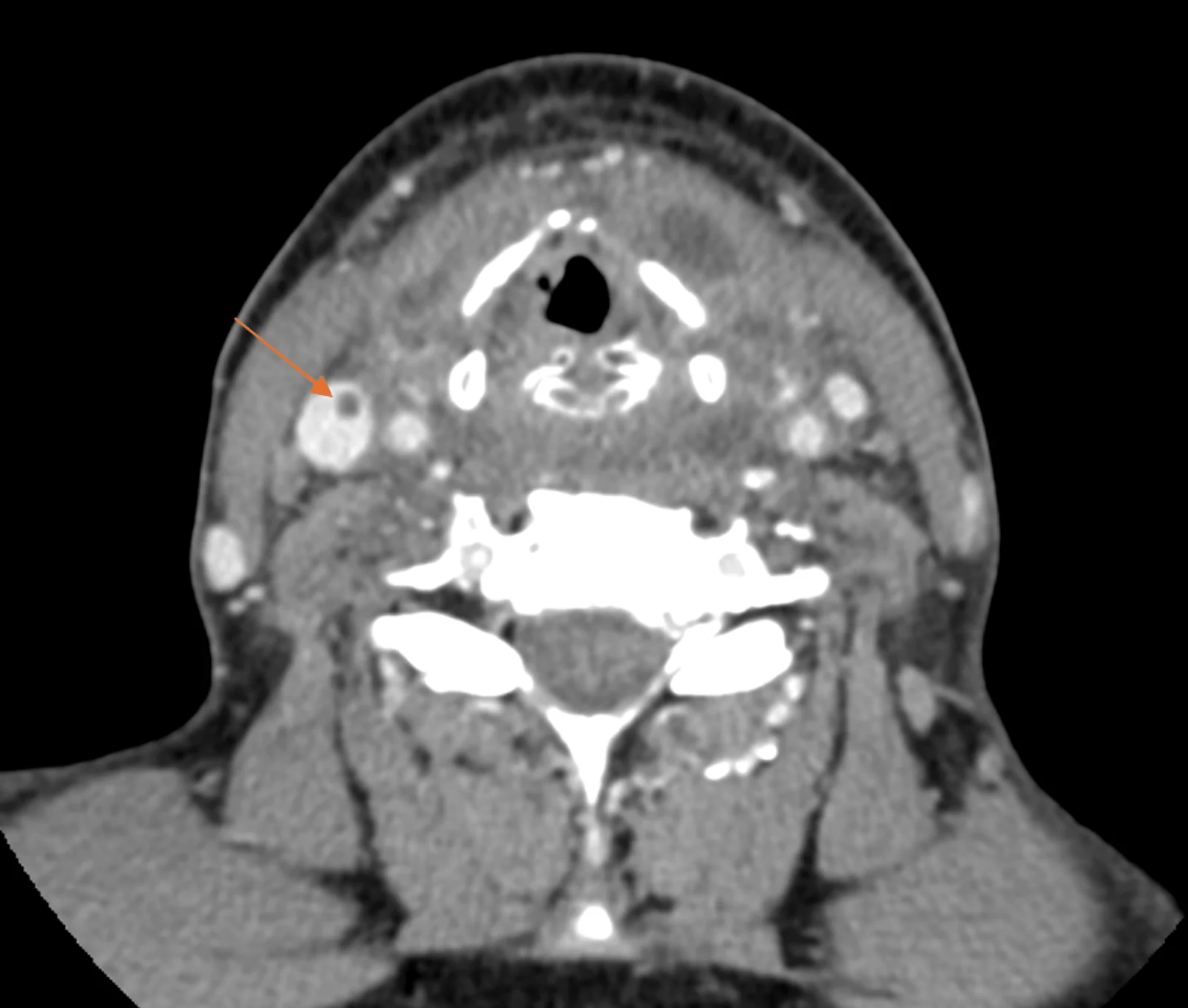
Computed tomography of the neck (axial view). Computed tomographic images of the neck demonstrating a non-occluding thrombus, identified by the arrow, measuring approximately 4 mm in thickness and 15 mm in length within the right internal jugular vein.

**Table 1:** Laboratory findings on initial presentation to the emergency department. CRP: C-reactive protein

Test	Result	Units	Reference range
Serum sodium	136	mmol/L	133-146
Serum potassium	4.0	mmol/L	3.5-5.3
Serum urea	9.0	mmol/L	2.5-7.8
Serum creatinine	130	µmol/L	45-84
CRP	565	mg/L	0-5
Haemoglobin	148	g/L	125-165
White cell count	22.9	10⁹/L	4.0-11
Platelet count	325	10⁹/L	150-400
Neutrophils	20.3	10⁹/L	2-7.5
Lymphocytes	1.0	10⁹/L	1.50-4.00
Monocytes	1.4	10⁹/L	0.2-0.8
Eosinophils	0.1	10⁹/L	0-0.4
Basophils	0.0	10⁹/L	0-0.1

She underwent incision and drainage of the abscess with insertion of a corrugated drain from the wound site and was commenced on heparin for anticoagulation. She completed a 10-day course of IV ceftriaxone and metronidazole whilst an inpatient. Following discharge, she completed a further two-week course of oral co-amoxiclav and metronidazole, with anticoagulation converted to apixaban for a total of three months.

Despite completing a further 10-day course of clindamycin, she re-presented three weeks after initial hospital discharge with persistent fever, worsening shortness of breath, and purulent neck wound discharge. Laboratory results showed leucocytes of 13.0 × 10⁹/L and CRP of 132 mg/L; full results are in Table 2. Further CT imaging revealed bilateral multiloculated empyema with mediastinal collections, as seen in Figure 2. Her parapharyngeal abscess showed almost complete resolution.

**Table 2:** Laboratory findings on readmission three weeks after initial discharge. CRP: C-reactive protein

Test	Result	Units	Reference range
Serum sodium	134	mmol/L	133-146
Serum potassium	4.0	mmol/L	3.5-5.3
Serum urea	2.0	mmol/L	2.5-7.8
Serum creatinine	41	µmol/L	45-84
CRP	132	mg/L	0-5
Haemoglobin	106	g/L	125-165
White cell count	13.1	10⁹/L	4.0-11
Platelet count	578	10⁹/L	150-400
Neutrophils	7.6	10⁹/L	2-7.5
Lymphocytes	4.1	10⁹/L	1.50-4.00
Monocytes	1.4	10⁹/L	0.2-0.8
Eosinophils	0.0	10⁹/L	0-0.4
Basophils	0.0	10⁹/L	0-0.1

**Figure 2:**
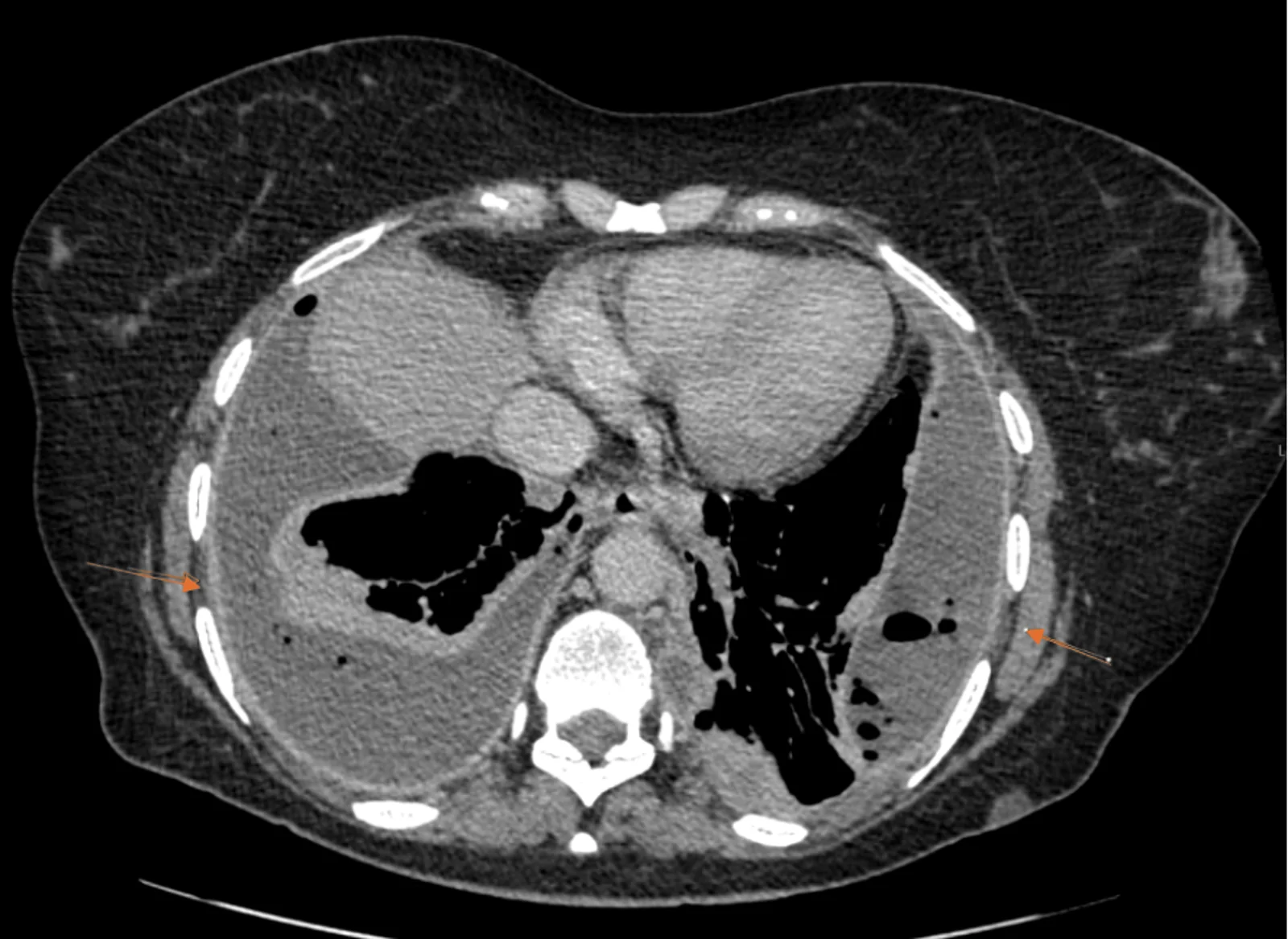
Computed tomography of the chest (axial view). Axial computed tomographic images of the chest taken at 1.25 mm intervals with intravenous contrast. There is bilateral multiloculated empyema with thick, enhancing walls, internal air locules, and air-fluid levels, with the right side appearing worse than the left. Bilateral changes identified by arrows.

A transthoracic echocardiogram was performed to assess for infective endocarditis, which confirmed no obvious vegetations and good systolic function. She was commenced on IV piperacillin-tazobactam and readmitted for ongoing management. A right-sided 18-French Seldinger chest drain was inserted, which drained purulent fluid, and treatment-dose enoxaparin was commenced alongside metronidazole to cover for mediastinitis. A further left-sided drain was also inserted. Pleural fluid did not show any growth on routine or broth culture. Despite bilateral drains, repeat CT after six days showed slight reduction in pleural collections but otherwise broadly static appearances (Figure 3). Findings were discussed with the local tertiary thoracic centre, which accepted for transfer for ongoing management.

**Figure 3:**
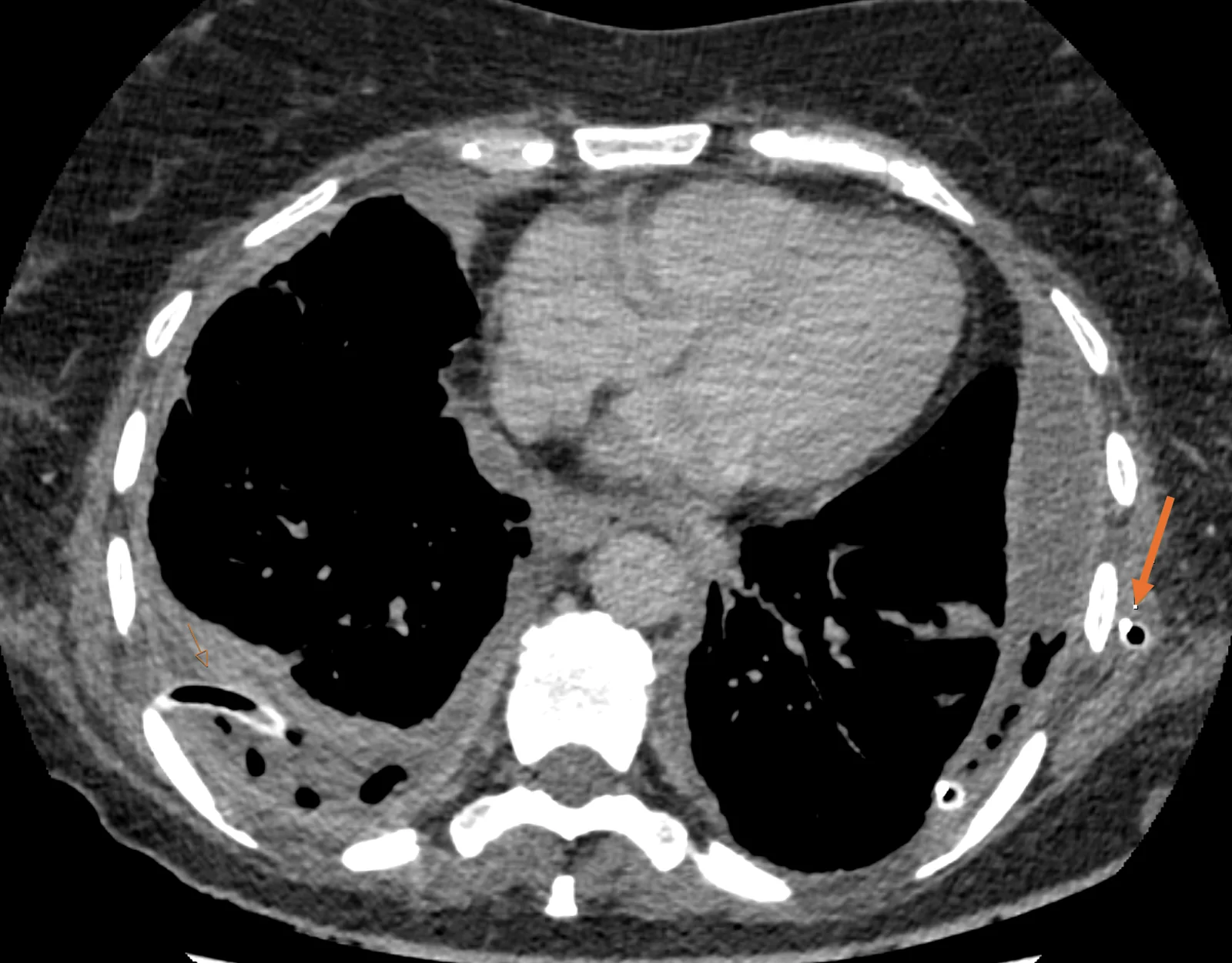
Repeat computed tomography of the chest with chest drain in situ (axial view). Computed tomographic images of the chest performed six days after Figure 1 images. There are bilateral chest drains present (left-sided drain only visible in this static image) with slight reduction in the size of the bilateral empyema but otherwise a broadly static appearance. Arrow identifying left-sided chest drain placement.

Following transfer, she underwent video-assisted thoracoscopic surgery (VATS) washout of the right empyema with three chest drains placed. Left-sided effusion improved with percutaneous drainage and did not require VATS washout. Drains were removed sequentially over the following days, and post-drain removal, chest x-rays were stable. Repeat CT imaging following VATS showed considerable improvement (Figure 4). Histology sent from pleural biopsy following procedure was consistent with empyema. She received a prolonged 21-day course of IV piperacillin-tazobactam and metronidazole as per microbiology input and was later stepped down to oral co-amoxiclav upon discharge for a total of four weeks post-surgery. A CT pulmonary angiogram, performed due to tachycardia, excluded pulmonary embolism.

**Figure 4:**
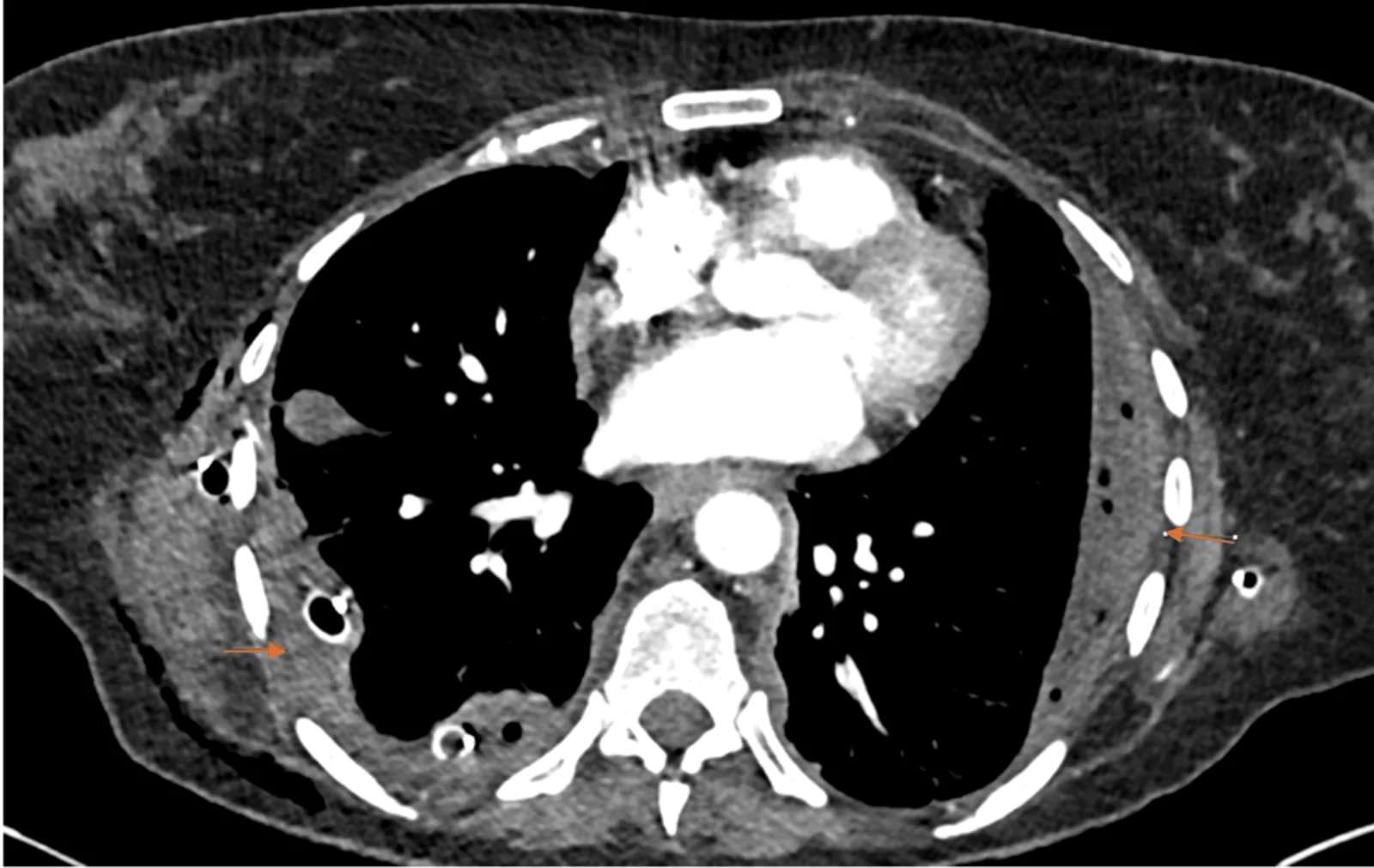
Computed tomographic pulmonary angiography (axial view). Computed tomographic angiography of the chest performed following video-assisted thoracoscopic surgery for right empyema. Images demonstrate reduction in bilateral empyema, as identified by arrows. Bilateral chest drains remain in situ.

She was discharged with a 20-day course of antibiotics and advice to hold her regular adalimumab injection until the course was completed. Subsequent clinic follow-up and interval chest x-rays showed improvement in symptoms and resolution of left pleural thickening, with the right significantly improved. Despite considerable improvement, she continued to experience shortness of breath on minimal exertion several months after discharge. One year later, she remained well with no further complications but reported ongoing impact of breathlessness on daily activities.

## Discussion

LS is a rare but life-threatening condition that usually affects young, immunocompetent individuals following oropharyngeal infection [4]. The syndrome often results in pulmonary involvement, with the literature commonly citing infection developing from septic emboli, cavitating nodules, or pleural effusions [10]. Unilateral empyema is a relatively common finding; however, the progression to bilateral, multiloculated empyema, as seen in this patient, is uncommon and represents a severe complication [11].

Two cases of bilateral empyema in the context of LS have been published to date, both of which were in immunocompetent individuals [2,11]. This suggests bilateral empyema is a clinically rare yet life-threatening complication, particularly in immunocompromised individuals with a 1.5-fold increased risk of adverse outcomes [12]. The pathophysiology in this case likely reflects septic embolisation from the thrombosed jugular vein to the pulmonary vasculature, with subsequent pleural infection. Despite no bacterial growth from pleural fluid cultures being identified, culture-negative empyema in the context of prior antibiotic exposure is well recognised and should not delay aggressive management when radiological and clinical features are convincing [13]. Furthermore, documented LS cases in recent years have increased, with the suggestion that this is partly due to less aggressive initial antibiotic therapy and wider antibiotic resistance [4,14]. As such, aggressive, tailored, where possible, antibiotic therapy should be commenced promptly when clinical suspicion arises.

Despite antibiotic therapy, treatment options for complex empyema usually require some form of surgical drainage as antibiotics alone are insufficient [13]. However, evidence shows that patients initially treated with percutaneous drainage, as in this case, often had an increased need for repeat intervention compared with thoracic surgery drainage alone [15-17]. Therefore, the gold standard of multiloculated empyema drainage in complex patients not responding to medical treatment remains pleural decortication, preferably via VATS [13,18]. This case highlights the importance of early specialist thoracic input, as management required escalation due to the complex, non-resolving nature of the disease. Furthermore, bilateral disease poses additional physiological burden and increases the risk of long-term pulmonary complications. This is evidenced by the patient’s ongoing exertional dyspnoea several months later, illustrating the significant morbidity associated with such cases.

The most common pathological organism associated with LS is *F. necrophorum*, an anaerobic, gram-negative bacillus [4]. It was debated whether cases that do not isolate *F. necrophorum* are truly LS; however, a range of alternative, rarer pathogens have been recently documented and widely accepted as causes of LS [5]. In this case, S. pyogenes was isolated from the deep neck abscess, providing a plausible causative organism, and the subsequent clinical syndrome remained characteristic of LS. This highlights the importance of recognising that LS is a clinical syndrome rather than an infection caused by a single organism. In particular, clinicians should remain alert to alternative pathogens when managing patients with severe oropharyngeal infection complicated by IJV thrombosis and systemic infection.

Classically, LS affects young, healthy individuals, with reports in immunocompromised individuals limited [4]. The patient in this case was receiving adalimumab for rheumatoid arthritis, a tumour necrosis factor-alpha inhibitor associated with increased susceptibility to serious bacterial infections [19]. This may have modified the clinical trajectory, where metastatic complications evolved weeks after initial presentation, possibly as a result of incomplete resolution of infection. Moreover, it could be considered that immunosuppression contributed to a high septic burden or delayed eradication of bacteraemia, which resulted in a more unusual presentation of bilateral disease. Despite this, the contribution of immunosuppression to disease severity remains uncertain. Therefore, this highlights the importance of maintaining a low threshold for repeat imaging and prolonged antimicrobial therapy, particularly in immunosuppressed patients despite favourable early responses.

The role of anticoagulation in LS remains debated, with uncertainty due to potential bleeding risk. Despite lack of controlled evidence suggesting its benefit, observational data suggest potential benefit in preventing thromboembolic complications, and that use of anticoagulants prior to complications developing may be a favourable prognostic factor [20]. In immunocompromised patients with extensive thrombosis and metastatic spread, the risk-benefit balance may favour anticoagulation, as in this case, but decisions must remain individualised.

## Conclusions

This case highlights the potential for progression of LS to complex thoracic involvement, including bilateral multiloculated empyema, despite appropriate initial therapy. Early recognition, with tailored antimicrobial therapy and surgical intervention, was essential for a successful outcome. Immunosuppression secondary to rheumatoid arthritis may have contributed to disease severity in this case, but its impact remains uncertain. The unusual causative organism also highlights that LS should be considered despite the absence of *F. necrophorum*, and early recognition should instead rely on characteristic clinical and radiological features.
